# Fatness-Associated FTO Gene Variant Increases Mortality Independent of Fatness – in Cohorts of Danish Men

**DOI:** 10.1371/journal.pone.0004428

**Published:** 2009-02-13

**Authors:** Esther Zimmermann, Sofia I. I. Kring, Tina L. Berentzen, Claus Holst, Tune H. Pers, Torben Hansen, Oluf Pedersen, Thorkild I. A. Sørensen, Tine Jess

**Affiliations:** 1 Institute of Preventive Medicine, Copenhagen University Hospitals, Centre for Health and Society, Copenhagen, Denmark; 2 Center for Biological Sequence Analysis, Technical University of Denmark, Lyngby, Denmark; 3 Steno Diabetes Center, Copenhagen, Denmark; Universidad Nacional Mayor de San Marcos, Peru

## Abstract

**Background:**

The A-allele of the single nucleotide polymorphism (SNP), rs9939609, in the FTO gene is associated with increased fatness. We hypothesized that the SNP is associated with morbidity and mortality through the effect on fatness.

**Methodology/Principal Findings:**

In a population of 362,200 Danish young men, examined for military service between 1943 and 1977, all obese (BMI≥31.0 kg/m^2^) and a random 1% sample of the others were identified. In 1992–94, at an average age of 46 years, 752 of the obese and 876 of the others were re-examined, including measurements of weight, fat mass, height, and waist circumference, and DNA sampling. Hospitalization and death occurring during the following median 13.5 years were ascertained by linkage to national registers. Cox regression analyses were performed using a dominant effect model (TT vs. TA or AA). In total 205 men died. Mortality was 42% lower (p = 0.001) with the TT genotype than in A-allele carriers. This phenomenon was observed in both the obese and the randomly sampled cohort when analysed separately. Adjustment for fatness covariates attenuated the association only slightly. Exploratory analyses of cause-specific mortality and morbidity prior to death suggested a general protective effect of the TT genotype, whereas there were only weak associations with disease incidence, except for diseases of the nervous system.

**Conclusion:**

Independent of fatness, the A-allele of the *FTO* SNP appears to increase mortality of a magnitude similar to smoking, but without a particular underlying disease pattern barring an increase in the risk of diseases of the nervous system.

## Introduction

Various studies have shown that a series of common single nucleotide polymorphisms (SNPs) in the first intron of the FTO gene is associated with higher body-mass index (BMI) and risk of obesity in European cohorts [1]–[4]. These SNP's are in tight linkage disequilibrium so studies of only one of them will convey the effect. Since fatness, and especially abdominal fatness, is associated with increased mortality [5]–[7], we hypothesized that the *FTO* SNP, rs9939609, T/A with a minor A-allele frequency in Caucasians of 0.45, is associated with mortality through its association with fatness-related diseases (GenBank accession no.: NT_010498). We therefore investigated the association between this SNP and morbidity and mortality with and without adjustment for fatness.

## Methods

### Study population

**Figure 1:**
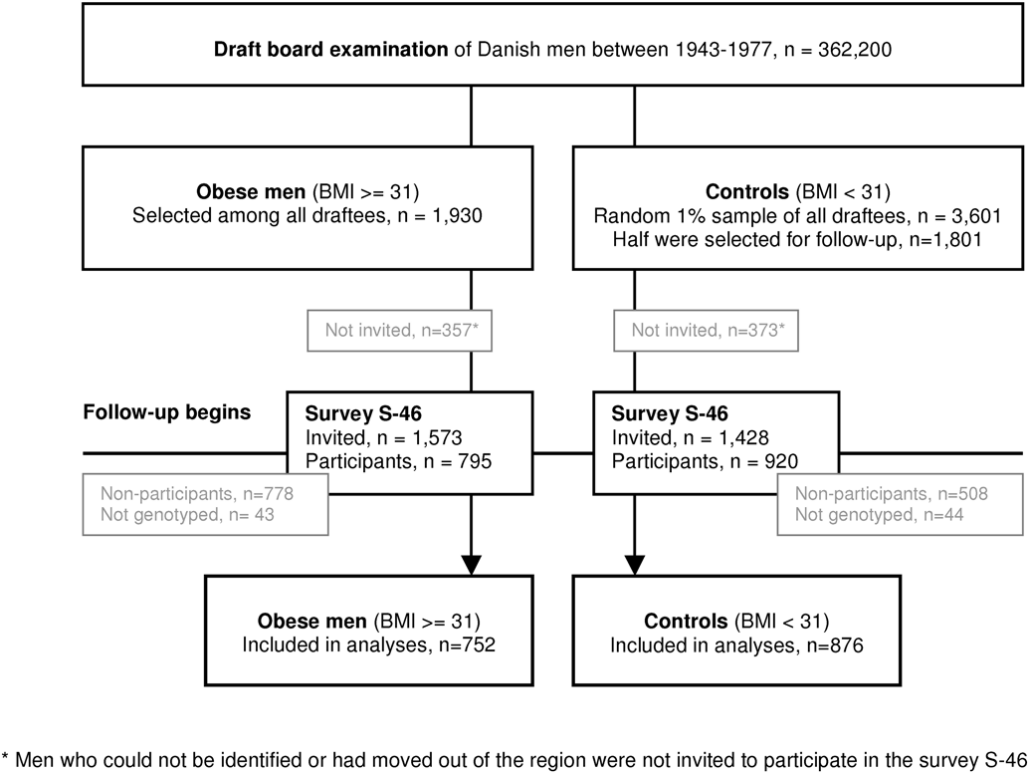
Participation flow chart from draft board examination.

Among 362,200 Caucasian men examined at the mean age of 20 years at the draft boards in Copenhagen and its surroundings during 1943–77, a randomly selected group of one in every hundred men (n = 3,601) and all obese men (n = 1,930) were manually identified (referred to as survey S-20) [8]; [9]. Obesity was defined as 35% overweight relative to a local standard in use at the time, corresponding to a BMI≥31.0 kg/m^2^, which proved to be above the 99^th^ percentile. All obese and half of the random sample, still living in the region, were invited to a follow-up survey in 1992–94. Due to the long sampling period (1943–1977) the age range at follow-up was wide, resulting in a mean age of 46 years (referred to as survey S-46) [4]; [10]. The participation rate was 50.5% among the obese and 64.4% among the randomly sampled, equivalent to 795 obese and 920 randomly sampled men attending the examination (figure 1), which included sampling of blood, from which DNA was extracted. Genotyping of *FTO* rs9939609 (Taqman allelic discrimination; KBiosciences, Cambridge, UK) was successful in 96% of the samples - 752 obese and 876 randomly sampled men - with an error rate of 0.72% calculated from 553 duplicate samples in men that additionally had blood drawn at a mean age of 49 years (referred to as S-49). In case of genotype discrepancy, the genotype from S-49 was used, since the quality of these samples was judged to be of highest quality. Population stratification may occur due to differences in allele frequencies between the obese and randomly sampled men owing to systematic differences in ancestry rather than association of genes with the response variable. However, our cohort study design of Danish Caucasian men where the randomly sampled men come from the same population in which the obese men were identified effectively prevents population stratification. This is reflected in the genotype distributions of the sample, which obeyed Hardy-Weinberg equilibrium (p = 0.27). The attrition of the groups over time implied that the segment of subjects that were followed up and genotyped represent a population of approximately 175,000 men (represented by the 0.5% random sample of 876 men (200*876 = 175.200)) and the originally obese men in this population. The median BMI at time of draft board examination was 21.3 (range: 15.7–30.9) among randomly sampled men included in the present study compared to a median BMI of 21.4 (range: 15.7–30.9) for randomly sampled men excluded from this study (for any reason). Similarly, for obese men included in the present study the median BMI at draft board examination was 32.5 (range: 31.0–51.8) versus 32.8 (range: 31.0–45.9) among obese men not included in the present study (for any reason).

Objective measures of weight and height were obtained at S-20 and S-46, and additionally, waist circumference was measured at S-46. BMI was calculated as weight in kilograms divided by the square of height in meters. Total body fat mass was assessed by bioimpedance at S-46, and fat body-mass index (fat-BMI) was calculated as weight of body fat mass in kilograms divided by the square of height in meters. Bioimpedance has proved to be reasonable accurate for assessing body composition (fat mass and fat free mass) in cohort studies [11].

The Danish Civil Registration System was established on April 2, 1968 [12]. All Danish residents alive or born thereafter have been assigned a unique identification number, referred to as their CPR number. Information on vital status and occurrence of disease was obtained by linking the subjects' CPR number to the Civil Registration System and the National Hospital Discharge Register [13], respectively, until October 31, 2007. Information on causes of death was obtained by linkage to the National Cause of Death Register [14] until 2007. Using an explorative approach, the sequences of diseases that had contributed to death according to the death certificate were investigated. In case of co-morbidity, a subject was only allowed to appear in each disease category once, but in several disease categories simultaneously. An additional approach was undertaken to investigate the occurrence of disease in the year preceding death in men who died during follow-up. Diseases occurring in this year convey supplementary information on diseases that may have contributed to death. The incidence of diseases emerging after the S-46 was analyzed in all men as time to first hospitalization following the survey as recorded in the National Hospital Discharge Register. The causes of death and hospital discharge diagnoses were classified according to the International Classification of Diseases, Eighth Revision (ICD-8) before 1994 and the Tenth Revision (ICD-10) thereafter. The coverage of each register is high, with virtually every event recorded [13]; [14].

### Ethics statement

The Danish Data Protection Agency and the Ethical Committees of Copenhagen and Frederiksberg municipalities approved the study, which was in accordance with the Helsinki Declaration II. All participants signed written consent before participating.

### Statistical analyses

Our study sample represents the entire BMI distribution through the random sample and with the right end of the distribution being richly represented. Therefore, the data actually consists of a cohort with two nested sub-cohorts based on the different sampling fraction across the range of BMI at draft board examination. This design implies that the data can be analysed as combined cohort data [15]. Cox proportional-hazards regression was used to test our *a priori* hypothesis of an association between *FTO* genotype and all-cause mortality. Analyses were performed for the original obese and the randomly sampled cohorts separately and combined, with and without adjustment for the various fatness covariates. No statistical interactions between genotype and the original sampling groups with regard to associations to the outcomes were observed. We explored the pattern of cause-specific mortality and morbidity. The analyses were performed for the two sampling cohorts combined with adjustment for BMI S-20 and BMI S-46.

For each Cox proportional-hazards model the subjects were followed from date of entry into S-46 until date of death, censoring due to loss to follow-up, emigration, or end of follow-up at October 31, 2007, in the Civil Registration System. The largest group (the carriers of the A-allele) was used as reference, in order to obtain the most precise estimates. The underlying time-scale was age, with delayed entry from age at S-46, which maximizes comparability by age [16]. Results are presented as hazard ratios (HR) with 95% confidence intervals (CI), and p-values <0.05 were considered significant. Linearity of the covariates was tested against a restricted cubic spline with five knots [17], and if the assumption of linearity was not fulfilled the covariates were included as restricted cubic splines. For every Cox model, the proportional hazards assumption was controlled with a smoothed plot of scaled Schoenfeld residuals versus time. Likelihood ratio tests were used to estimate whether a model including both fatness-covariates and the *FTO* SNP enhanced model fit compared to a model including only fatness-covariates. All analyses were performed in STATA version 9.2 (Stata Corporation, Texas; www.stata.com).

## Results

Out of the total of 1,628 men included in the analyses, 205 men died during the median 13.5 years of follow-up. The genotype distribution differed as expected between the originally obese men (TT, 25.5%, TA, 47.6%; AA, 26.9%) and the randomly sampled men (TT, 32.7%; TA, 50.6%, AA, 16.8%), corresponding to a minor allele frequency of 0.51 in the obese group, 0.42 in the random sampled group and 0.46 combined. The obese were slightly younger than the other men due to the increasing prevalence of obesity during the recruitment period (Table 1). The odds ratio for having a BMI≥31.0 at time of draft board examination was 2.05 (95% CI, 1.55–2.71; p<5*10^−7^) for the AA genotype versus the TT genotype.

**Table 1:** Distribution of age and anthropometrics given as median and range of 1,628 Danish men according to genotype for *FTO* rs9939609

		TT	TA	AA
		N = 478	N = 801	N = 349
No of subjects	Obese	192	358	202
	Random sample	286	443	147
No of deaths	Obese	14	54	30
	Random sample	26	63	18
Age (yr) S-46	Obese	41 (34–66)	43 (33–73)	41 (33–75)
	Random sample	46 (34–71)	47 (34–73)	46 (34–67)
BMI (kg/m^2^) S-20	Obese	32.5 (31.0–42.8)	32.5 (31.0–45.4)	32.7 (31.0–51.8)
	Random sample	21.3 (15.9–29.4)	21.2 (15.7–30.9)	21.4 (16.3–29.7)
BMI (kg/m^2^) S-46	Obese	34.6 (24.3–63.7)	34.9 (19.9–51.5)	35.6 (25.2–59.6)
	Random sample	25.7 (18.0–36.5)	25.8 (16.2–45.1)	25.9 (19.4–41.9)
Waist (cm) S-46	Obese	115.5 (79.5–183.0)	116.8 (84.0–160.0)	117.0 (81.0–171.5)
	Random sample	92.2 (70.0–130.0)	93.0 (66.0–131.0)	93.2 (58.0–139.0)
Fat-BMI (kg/m^2^) S-46	Obese	11.8 (4.9–32.1)	12.0 (3.4–24.3)	12.4 (4.7–26.7)
	Random sample	6.3 (1.2–13.3)	6.4 (0.3–19.2)	6.2 (1.5–16.6)

Abbreviations: BMI, body mass index; fat-BMI, fat mass (kg)/height^2^ (m^2^); N, number of subjects; S-20, draftee population of median age 20; S-46, follow-up survey at median age 46

### All-cause mortality

The mortality in the obese group was, as expected, greater than in the randomly selected group, with a HR of 2.03 (95% CI, 1.50–2.75). A dominant effect of the A-allele (TT genotype versus TA and AA genotypes) gave the best fit to the mortality data as illustrated in Table 2. This was confirmed by likelihood ratio tests (LRT) where an additive effect of the gene (LRT, 2.65; P = 0.10), a dominant effect of the gene (LRT, 0.45; P = 0.50) and a recessive effect of the gene (LRT, 10.77; P = 0.001) were tested. A decreased mortality in men with the TT genotype compared to A-allele carriers was observed, both among obese and the randomly sampled, and since there was no interaction, a pooled estimate was made; HR, 0.58; 95% CI, 0.41–0.82 (Table 3). The association was attenuated only slightly and remained significant after adjustment for BMI at S-46, waist circumference and BMI at S-46, weight history (i.e. BMI at S-20 and S-46), and fat-BMI at S-46 (Table 3).

**Table 2:** *FTO* rs9939609 genotype distribution and all-cause mortality in 1,628 Danish men

	TT		TA	AA	
	HR	95% CI	HR	HR	95% CI
Obese	0.52	0.29–0.94	1.00	1.06	0.68–1.66
Random sample	0.63	0.40–0.99	1.00	1.02	0.60–1.73
Combined	0.59	0.41–0.84	1.00	1.05	0.75–1.48

Cox proportional hazards analysis with age as the underlying time scale and delayed entrance at age at time of blood sampling (S-46)

Abbreviations: HR, Hazard ratio; CI, Confidence interval

**Table 3:** *FTO* rs9939609 (genotype TT vs. TA and AA) and all-cause mortality in 1,628 Danish men

		Obese			Random sample			Pooled		
	N	HR	95% CIs	P value	HR	95% CIs	P value	HR	95% CIs	P value
Crude	1,628	0.51	0.29–0.90	0.02	0.62	0.40–0.97	0.04	0.58*	0.41–0.82	0.001
Adjusted for BMI S-46	1,626	0.52	0.29–0.91	0.02	0.61	0.39–0.95	0.03	0.58*	0.41–0.82	0.002
Adjusted for BMI and waist S-46	1,624	0.51	0.29–0.91	0.02	0.59	0.38–0.92	0.02	0.57*	0.40–0.81	0.002
Adjusted for BMI S-20 and S-46	1,626	0.53	0.30–0.94	0.03	0.61	0.39–0.96	0.03	0.59*	0.41–0.83	0.003
Adjusted for fat-BMI S-46	1,523	0.59	0.33–1.05	0.07	0.64	0.41–1.01	0.06	0.63*	0.44–0.90	0.01

Cox proportional hazards analysis with age as the underlying time scale and delayed entrance at age at time of blood sampling (S-46). There was no interaction between the genotypes and the original sampling variable (Random sample vs. obese)

Abbreviations: BMI, body mass index; fat-BMI, fat mass (kg)/height^2^ (m^2^); N, number of subjects with complete covariate information; HR, Hazard ratio; CI, Confidence interval; S-20, draftee population of median age 20; S-46, follow-up survey at median age 46

* p<0.05 for the likelihood ratio test (Likelihood ratio tests were used to estimate whether a model including both fatness-covariates and FTO rs9939609 fitted the data better than a model including only fatness-covariates)

### Exploratory study of disease pattern

Fat-BMI apparently had the strongest effect on the association between the genotypes and all-cause mortality (Table 3), but since data on fat-BMI was missing for 103 subjects, the cause-specific analyses were instead adjusted for BMI at S-20 and S-46. The results were essentially the same when fat-BMI was included as a covariate instead of BMI at S-20 and S-46. Table 4 shows the disease groups explored (ICD codes are available on request).

**Table 4:** *FTO* rs9939609 (genotype TT vs. TA and AA) and cause-specific mortality and morbidity in 1,628 Danish men adjusted for BMI S-20 and S-46

	TT			TA and AA	
**Infectious diseases**	**No**	**HR**	**95% CIs**	**No**	**HR**
Prevalent at time of death	3	1.17	0.30–4.60	7	1.00
Incident one year prior to death	4	0.55	0.18–1.63	17	1.00
First incidence	37	0.95	0.65–1.39	107	1.00
**Cancer**					
Prevalent at time of death	13	0.84	0.43–1.66	51	1.00†
Incident one year prior to death	14	0.68	0.38–1.25	48	1.00
First incidence	41	0.84	0.58–1.20	114	1.00
**Haematological diseases**					
Prevalent at time of death	0	0.00	-	3	1.00
Incident one year prior to death	3	0.55	0.16–1.92	14	1.00
First incidence	11	0.72	0.37–1.41	43	1.00
**Endocrine diseases**					
Prevalent at time of death	7	0.62	0.28–1.41	35	1.00
Incident one year prior to death	11	0.73	0.37–1.43	40	1.00
First incidence	64	0.73	0.55–0.96	238	1.00*
**Mental diseases**					
Prevalent at time of death	1	0.20	0.03–1.52	12	1.00*
Incident one year prior to death	2	0.33	0.07–1.48	13	1.00
First incidence	23	0.92	0.56–1.49	63	1.00
**Diseases of the nervous system**					
Prevalent at time of death	0	0.00	-	10	1.00
Incident one year prior to death	2	0.27	0.06–1.17	19	1.00*
First incidence	38	0.57	0.40–0.81	183	1.00*
**Circulatory diseases**					
Prevalent at time of death	20	0.70	0.42–1.15	74	1.00
Incident one year prior to death	20	0.73	0.44–1.21	70	1.00
First incidence	122	0.90	0.73–1.11	360	1.00
**Respiratory diseases**					
Prevalent at time of death	6	0.54	0.22–1.33	26	1.00
Incident one year prior to death	9	0.49	0.24–1.02	44	1.00*
First incidence	50	0.83	0.60–1.15	157	1.00
**Diseases of the digestive system**					
Prevalent at time of death	7	0.72	0.31–1.68	23	1.00
Incident one year prior to death	9	0.85	0.39–1.81	27	1.00
First incidence	92	0.94	0.74–1.20	246	1.00
**Diseases of the locomotion system**					
Prevalent at time of death	0	0.00	-	5	1.00
Incident one year prior to death	7	0.94	0.39–2.26	20	1.00
First incidence	152	1.12	0.92–1.36	347	1.00
**Diseases of the kidney/urinary system**					
Prevalent at time of death	2	0.59	0.12–2.82	8	1.00
Incident one year prior to death	6	0.66	0.26–1.63	22	1.00
First incidence	59	0.86	0.64–1.15	181	1.00
**Accidents/external causes**					
Prevalent at time of death	3	1.07	0.27–4.31	6	1.00
Incident one year prior to death	13	0.80	0.43–1.51	40	1.00
First incidence	213	1.06	0.90–1.25	512	1.00
**Other/unspecified diseases** ‡					
Prevalent at time of death	5	0.68	0.25–1.86	17	1.00
Incident one year prior to death	19	0.56	0.34–0.92	83	1.00*
First incidence	143	0.96	0.79–1.16	380	1.00

Cox proportional hazards analysis with age as the underlying time scale and delayed entrance at age at time of blood sampling (S-46). Cause of death information was not available in 21 men who died after December 31, 2006.

* p<0.05 for the likelihood ratio test (Likelihood ratio tests were used to estimate whether a model including both BMI S-20, BMI S-46 and FTO rs9939609 fitted the data better than a model including only BMI S-20 and BMI S-46)

† p<0.05 for interaction between genotype and the original sampling variable (random vs. obese); the interaction term is included in the model

‡ The category other/unspecified diseases includes benign neoplasms, diseases of the eye, skin and subcutaneous tissue, congenital malformation of the urinary system and symptoms, signs and abnormal clinical and laboratory findings, not otherwise classified.

Circulatory diseases and cancers accounted for the highest numbers of prevalent diseases at death, irrespective of genotype. For the majority of the disease categories, the estimates were lower for the TT genotype than for the A-allele carriers, although the estimates were not significantly different from 1.00 (Table 4). Remarkably, no individuals with the TT genotype died from or with diseases of the nervous system, whereas ten such cases were observed in the A-allele carriers.

Regarding diseases occurring during the year preceding death, the HR's were lower for the TT genotype (vs. TA and AA genotypes) in all categories, though not significantly different from 1.00 (Table 4).

The HRs for incidence of diseases during the follow-up after genotyping were in general very close to 1.00, indicating no difference in risk between the genotypes. However, a significantly lower risk was observed for endocrine diseases in TT versus A-allele carriers (HR, 0.73; 95% CI, 0.55–0.96) as well as for diseases of the nervous system (HR, 0.57; 95% CI, 0.40–0.82).

## Discussion

The present findings show a strong fatness-independent association between the *FTO* SNP, rs9939609, and all-cause mortality in middle-aged men of a magnitude similar to that of smoking [18], with a significantly decreased risk of dying in the TT genotype group compared with A-allele carriers. This was observed both in the random sample of the 362,200 Danish draftees and in the selected sub-sample of the most obese men in this population. Our *a priori* hypothesis – that the A-allele of the *FTO* SNP was associated with increased mortality and morbidity primarily through an effect on fatness – was thus not confirmed, as our results showed only a slight attenuation of the HR by adjusting for the fatness-covariates.

We observed that men with the AA genotype had a 2.05 increased odds of obesity compared with the TT genotype, which is higher than reported in previous studies [1]; [19]; [20]. This difference in odds ratios is most likely due to differences between the examined cohorts. Our cohort is characterized by a massive enrichment of the right tail of the BMI distribution, making it possible to demonstrate a stronger association compared to already published studies examining the effects of *FTO* on fatness. Men with the TT genotype had decreased mortality from most causes assessed. Our results suggest that the *FTO* SNP may affect particularly the nervous system, however, the number of cases with disease of the nervous system was small and could not alone account for the excess mortality observed among A-allele carriers.

In addition to death certificates providing the presumed sequence of diagnoses leading to death as well as other prevalent diseases, we also examinined inpatient registry data on diseases occurring close to time of death, thereby obtaining the best coverage of diagnoses contributing to death. As for prevalent diseases at death, assessment of these diseases showed lower risk estimates for almost all disease categories among individuals with the TT genotype. When analyzing incidence of diseases emerging earlier on (but still after genotyping), the TT genotype was less protective, hence suggesting that the genotype affects the subject's general ability to cope with disease once present rather than affecting susceptibility to developing disease. However, for diseases of the nervous and endocrine systems, a decreased occurrence was observed already at this stage.

The FTO gene is of ancient origin and conserved in vertebrates and marine algae [21]; [22]. Bioinformatics analyses suggests that *FTO* encodes a DNA demethylase, up-regulated by feeding and down-regulated by fasting [23] and studies suggest that the SNP influences cerebro-cortical insulin sensitivity [24] and lipolytic activity in adipose tissue of healthy individuals [25]. Our findings suggest that the *FTO* SNP may have much wider implications than the effect on fatness and the effects derived hereof. The observation of an increased mortality among A-allele carriers over the broad range of disease categories may translate into the hypothesis that the cellular effects of the SNP affects most organs, and particularly their ability to maintain or regenerate proper function when afflicted by various diseases. The positive association between A-allele carriers and disease of the nervous system also needs further investigation. It may be related to the high expression of *FTO* in fetal and adult brain tissues [1]; [2]; [21]; [23]. Due to the limited numbers of cases and the multiple testing, we emphasize that our findings are purely hypothesis generating. To test these hypothesis further research is warranted, both at the level of mapping disease patterns at a more detailed level than the organ specific disease categories performed here, and through further examination of the functional impact of the *FTO* SNP. Since the SNP is intronic it may exert functional effects through altered expression of *FTO*, or the observed effects may putatively be mediated via another gene.

There are several strengths of this study, but also limitations. It is based on a unique, well-defined large background study population of white men with the obese and random sample derived from the same quite homogenous population, hence eliminating population stratification. The complete sampling of an obese group facilitates valid estimation of the associations between the *FTO* SNP, fatness and morbidity and mortality, and hence also of fatness-independent associations. The inclusion of a randomly selected group gave us the possibility to reproduce our estimates as well as to study the broad range of BMI when combining the groups. The accessibility of complete register-based data on morbidity and mortality is an asset of the study, although the clinical routine procedure generating the information requires cautious interpretation. The sample size may seem small for a genetic association study, but this apparent limitation is counteracted by the fact that the random sample is representative of 175,000 men, among whom the obese participants represents the most extreme range of fatness phenotypes in the same population, except for the possible selective attrition before S-46, where the genotyping was performed. The obese group exhibited the expected excess mortality relative to the randomly sampled group. The finding that circulatory diseases and cancers were the categories with the largest number of cases, further confirmed that the sample was representative of the general male population, since these are listed as main causes of death in the Danish male population [26]. Further, we investigated morbidity and mortality in middle-aged men, hence, we cannot conclude on the *FTO* SNP's impact on disease-risk in childhood or young adulthood. The major limitation of the study is that the number of deaths is too small, and the information about the diseases leading to death too crude, to conduct a thorough disease-specific genotype-phenotype investigation. Given these limitations, analyses of the reasons for the excess mortality obviously need to be explored in other populations of both men and women.

In conclusion, the *FTO* SNP, rs9939609, may have a fatness-independent impact on mortality and morbidity leading to death, with a general lower risk in the TT genotype compared with A-allele carriers.
